# Draft Genome Sequences of 48 Vancomycin-Resistant Enterococcus faecium Strains Isolated from Inpatients with Bacteremia and Urinary Tract Infection

**DOI:** 10.1128/MRA.00222-19

**Published:** 2019-04-11

**Authors:** Zulema Udaondo, Thidathip Wongsurawat, Piroon Jenjaroenpun, Courtney Anderson, James Lopez, Meera Mohan, Ruslana Tytarenko, Brian Walker, Intawat Nookaew, David Ussery, Atul Kothari, Se-Ran Jun

**Affiliations:** a Department of Biomedical Informatics, University of Arkansas for Medical Sciences, Little Rock, Arkansas, USA; b Division of Infectious Diseases, University of Arkansas for Medical Sciences, Little Rock, Arkansas, USA; c Myeloma Center, University of Arkansas for Medical Sciences, Little Rock, Arkansas, USA; University of Arizona

## Abstract

Vancomycin-resistant Enterococcus faecium (VREfm) is a major cause of nosocomial infections of the bloodstream and urinary tract. Here, we report the draft genome sequences of 48 vancomycin-resistant E. faecium isolates recovered from inpatients exhibiting clinical signs of bacteremia at the University of Arkansas for Medical Sciences (UAMS).

## ANNOUNCEMENT

Enterococcus faecium is an opportunistic environmental bacterium with an outstanding adaptive capacity to evolve and transfer antimicrobial-resistant determinants (1). E. faecium can cause persistent infections because of inherent and acquired resistance to common antibiotics, such as ampicillin and vancomycin (2). Vancomycin resistance can be conferred by different acquired gene clusters (*vanA*, *vanB*, *vanD*, *vanE*, *vanG*, *vanL*, *vanM*, and *vanN*), with *vanA* and *vanB* being the most common (3, 4). Decreasing the intestinal domination of vancomycin-resistant Enterococcus faecium (VREfm) in hospitalized patients and reducing the rate of patient-to-patient transmission are still challenging goals. To better understand the mechanisms of antibiotic resistance and the dynamics of transmission of this species in the hospital setting, we sequenced and analyzed 48 VREfm isolates collected from the University of Arkansas for Medical Sciences (UAMS) Hospital.

The VREfm isolates presented here were identified from positive blood and urine cultures (24 each). Identification and antimicrobial susceptibility tests were performed in the UAMS Clinical Microbiology Laboratory using standard microbiology techniques. More specifically, blood cultures were processed on the BacT/Alert 3D (bioMérieux) system. Positive blood cultures were subcultured on blood agar plates. Isolated colonies were then used for identification and susceptibility testing using the Vitek MS and Vitek 2 systems. Vancomycin resistance was confirmed using Etests (bioMérieux). Urine cultures were processed similarly using blood agar plates for the isolation of colonies. Antimicrobial susceptibility test results were interpreted using the M100 CLSI standards (5). Microbial DNA was extracted from pure growth of VREfm. Isolated colonies on the blood agar plates were picked and resuspended into a DNA/RNA shield collection and lysis tube (Zymo Research, Irvine, CA). Genomic DNA was extracted from the tube using the Quick-DNA fungal/bacterial kit (Zymo Research, Irvine, CA). The purity of extracted DNA was determined using a NanoDrop spectrophotometer by measuring the *A*_260/280_ and *A*_260/230_ ratios. DNA integrity and quantity were determined using an Agilent 2200 TapeStation and a Qubit 3.0 assay, respectively. Paired-end 150-bp libraries were constructed using the Kapa HyperPlus kit (Roche) with enzymatic fragmentation for 10 min. The resulting genomic libraries of the 48 E. faecium isolates were sequenced using the Illumina NextSeq 550 platform at the University of Arkansas for Medical Sciences Myeloma Center.

Adapters from the reads were trimmed using default parameters of fastp v0.19.5 (6) software, and poor-quality bases were removed using Trimmomatic v0.38 (7) with the following parameters: headcrop, 15; leading, 20; trailing, 20; slidingwindow, 5:20; and minlen, 50. The quality of the pre- and postprocessed reads was analyzed using the FastQC tool v0.11.8 (8). The resulting high-quality reads were assembled *de novo* using SPAdes v3.13.0 (9) with the “error-correction” and “careful” options; k-mer sizes of 21, 33, 55, and 77; and a minimum contig size of 500 bp. The draft genome sequences were checked for quality using default settings in QUAST v5.0.2 (10).

The genome sequences were submitted for annotation to the NCBI Prokaryotic Genome Annotation Pipeline (PGAP) (11) using the default parameters. All 48 isolates were screened for vancomycin resistance genes, verifying the presence of the *vanA* gene in all genomes in the study.

### Data availability.

This whole-genome shotgun project has been deposited at DDBJ/ENA/GenBank under the accession numbers detailed in Table 1. The versions described in this announcement are the first ones. The GenBank and BioSample accession numbers are given in Table 1. Raw sequence data for this study are available at the Sequence Read Archive under accession number PRJNA520878.

**TABLE 1:** Assembly metrics and accession numbers for 48 E. faecium isolates

Isolate name	No. of contigs	Total length (bp)	GC (%)	No. of reads	*N*_50_ (bp)	BioSample no.	GenBank accession no.	No. of ORFs^*a*^
UAMSEF_02	193	2,910,878	37.80	6,839,636	36,818	SAMN10869073	SEYV00000000	3,046
UAMSEF_03	198	2,911,646	37.80	7,114,878	35,022	SAMN10869074	SEYU00000000	3,044
UAMSEF_04	204	2,909,821	37.80	4,688,460	35,022	SAMN10869075	SEYT00000000	3,047
UAMSEF_05	203	2,911,291	37.80	4,881,024	34,261	SAMN10869076	SEYS00000000	3,054
UAMSEF_06	199	2,915,048	37.80	5,499,612	35,022	SAMN10869077	SEYR00000000	3,057
UAMSEF_07	200	2,921,117	37.79	5,228,072	33,402	SAMN10869078	SEYQ00000000	3,058
UAMSEF_10	204	3,029,727	37.66	6,167,912	35,022	SAMN10869079	SEYP00000000	3,208
UAMSEF_11	209	3,029,905	37.66	7,152,790	35,022	SAMN10869080	SEYO00000000	3,209
UAMSEF_12	209	3,030,170	37.66	7,191,884	35,022	SAMN10869081	SEYN00000000	3,208
UAMSEF_13	208	3,031,358	37.66	5,108,778	35,022	SAMN10869082	SEYL00000000	3,210
UAMSEF_14	207	3,033,907	37.66	5,789,340	35,022	SAMN10869083	SEYK00000000	3,228
UAMSEF_15	208	3,030,699	37.66	5,022,872	34,261	SAMN10869084	SEYJ00000000	3,218
UAMSEF_16	213	3,029,394	37.66	5,993,870	34,261	SAMN10869085	SEYI00000000	3,214
UAMSEF_17	219	3,036,737	37.66	6,044,830	34,261	SAMN10869086	SEYH00000000	3,227
UAMSEF_18	208	3,028,651	37.66	5,560,272	34,261	SAMN10869087	SEYG00000000	3,212
UAMSEF_19	213	3,030,152	37.66	7,403,936	34,261	SAMN10869088	SEYF00000000	3,213
UAMSEF_21	198	2,891,508	37.75	6,655,902	32,987	SAMN10869089	SEYE00000000	3,031
UAMSEF_22	159	2,749,498	37.95	6,867,326	42,992	SAMN10869090	SEYD00000000	2,857
UAMSEF_23	123	2,758,039	37.96	6,408,904	53,183	SAMN10869091	SEYC00000000	2,849
UAMSEF_24	40	2,770,238	38.23	7,386,962	246,941	SAMN10869092	SEYB00000000	2,792
UAMSEF_25	195	2,928,194	37.71	8,757,500	33,213	SAMN10869093	SEYA00000000	3,068
UAMSEF_26	29	2,664,140	37.94	4,900,272	136,618	SAMN10869094	SEXZ00000000	2,721
UAMSEF_27	222	2,902,431	37.71	5,726,412	31,483	SAMN10869095	SEXY00000000	3,058
UAMSEF_28	172	2,739,636	37.88	6,372,790	32,818	SAMN10869096	SEXX00000000	2,835
UAMSEF_29	199	2,879,498	37.75	5,414,500	31,547	SAMN10869097	SEXW00000000	3,036
UAMSEF_30	225	3,101,010	37.66	10,543,326	35,053	SAMN10869098	SEXV00000000	3,306
UAMSEF_31	229	3,096,967	37.66	6,861,760	33,476	SAMN10869099	SEXU00000000	3,313
UAMSEF_32	243	3,052,772	37.61	6,171,754	30,771	SAMN10869100	SEXT00000000	3,234
UAMSEF_33	202	2,880,836	37.74	7,659,930	31,547	SAMN10869101	SEXS00000000	3,030
UAMSEF_34	193	2,918,402	37.80	6,272,742	35,022	SAMN10869102	SEXR00000000	3,073
UAMSEF_35	173	2,849,207	37.74	6,435,630	40,697	SAMN10869103	SEXQ00000000	2,981
UAMSEF_36	191	2,845,779	37.74	6,167,138	40,603	SAMN10869104	SEXP00000000	2,996
UAMSEF_37	221	2,975,344	37.74	7,491,600	34,261	SAMN10869105	SEXO00000000	3,161
UAMSEF_38	187	2,938,030	37.62	6,602,994	43,049	SAMN10869106	SEXN00000000	3,097
UAMSEF_39	217	2,943,498	37.59	5,659,958	32,692	SAMN10869107	SEXM00000000	3,123
UAMSEF_40	172	2,846,352	37.74	6,122,006	46,310	SAMN10869108	SEXL00000000	2,972
UAMSEF_41	204	2,855,455	37.76	7,229,612	38,111	SAMN10869109	SEXK00000000	2,997
UAMSEF_42	237	3,050,710	37.61	6,845,894	30,771	SAMN10869110	SEXJ00000000	3,235
UAMSEF_43	185	2,847,828	37.73	7,869,526	40,603	SAMN10869111	SEXI00000000	2,978
UAMSEF_44	215	2,904,552	37.72	6,379,180	31,234	SAMN10869112	SEXH00000000	3,056
UAMSEF_45	185	2,845,170	37.78	6,850,388	37,403	SAMN10869113	SEXG00000000	2,971
UAMSEF_46	215	2,739,355	37.89	6,831,284	36,762	SAMN10869114	SEXF00000000	2,837
UAMSEF_47	216	2,927,294	37.74	6,728,684	34,275	SAMN10869115	SEYM00000000	3,073
UAMSEF_48	177	2,847,481	37.73	8,622,512	41,250	SAMN10869116	SEXE00000000	2,977
UAMSEF_49	226	2,993,736	37.58	8,560,860	32,693	SAMN10869117	SEXD00000000	3,170
UAMSEF_50	171	2,831,453	37.75	6,264,722	44,556	SAMN10869118	SEXC00000000	2,960
UAMSEF_51	174	2,848,308	37.74	8,268,752	43,050	SAMN10869119	SEXB00000000	2,975
UAMSEF_52	209	2,898,662	37.76	6,504,814	32,904	SAMN10869120	SEXA00000000	3,055

a ORFs, open reading frames.
